# Knockdown of* SIRT1* Suppresses Bladder Cancer Cell Proliferation and Migration and Induces Cell Cycle Arrest and Antioxidant Response through FOXO3a-Mediated Pathways

**DOI:** 10.1155/2017/3781904

**Published:** 2017-09-25

**Authors:** Qingxuan Hu, Gang Wang, Jianping Peng, Guofeng Qian, Wei Jiang, Conghua Xie, Yu Xiao, Xinghuan Wang

**Affiliations:** ^1^Department of Urology, Zhongnan Hospital of Wuhan University, Wuhan, China; ^2^Department of Endocrinology, The First Affiliated Hospital of Zhejiang University, Hangzhou, China; ^3^Medical Research Institute, Wuhan University, Wuhan, China; ^4^Center for Medical Science Research, Zhongnan Hospital of Wuhan University, Wuhan, China; ^5^Department of Radiation and Medical Oncology, Zhongnan Hospital of Wuhan University, Wuhan, China; ^6^Department of Biological Repositories, Zhongnan Hospital of Wuhan University, Wuhan, China

## Abstract

Bladder cancer (BCa) is one of the most common tumors, but its underlying mechanism has not been fully clarified. Our transcriptome analysis suggested a close link of Sirtuins, Peroxisome Proliferator-Activated Receptor (PPAR), cell cycle regulation, reactive oxygen species (ROS) metabolism, and Forkhead Box Class O (FOXO) signaling pathway in BCa. SIRT1 is a key member of Sirtuins, playing important roles in aging and energy metabolism, which has been reported to be involved in various metabolic diseases and tumors. We observed that SIRT1 was upregulated in BCa tissues at both mRNA and protein levels. By establishing a* SIRT1*-knockdown BCa cell model, our results suggested that proliferation and viability were suppressed. Moreover, migration rate was inhibited as well, possibly via reduction of epithelial-mesenchymal transition (EMT). In addition, cell cycle arrest was significantly induced, consisting with strongly decreased proteins involved (CDK2/4/6). Furthermore, ROS production was slightly reduced, accompanied by increasing of antioxidant enzymes and total/acetylated FOXO3a. Consistently with our Path-net analysis, we observed no significant alteration of apoptosis in the* SIRT1*-knockdown BCa cells. Taken together, our results suggested that* SIRT1* deficiency in BCa cells could suppress cell viability by activating antioxidant response and inducing cell cycle arrest possibly via FOXO3a-related pathways.

## 1. Introduction

Bladder cancer (BCa), also named urinary bladder cancer, is one of the most common tumors ranking as the 9th leading cause of death worldwide [1, 2]. A horrible threat as it is to human health, its underlying mechanism, especially its metabolic alterations, has not been fully clarified yet [3–5].

Sirtuins, also known as Sir2-like proteins, are a family of NAD^+^-dependent deacetylases and ADP-ribosyltransferases [6], playing a vital role in aging [7]. Sir2 was first discovered in yeast via a model of replicative lifespan [8], and later it was shown that addition of an extra copy of the* SIR2* gene could extend replicative lifespan by 40% while deleting* SIR*2 shortened lifespan [9].

Mammals contain seven Sirtuins (SIRT1–7), which are categorized by their highly conserved central NAD^+^-binding and catalytic domain, termed the Sirtuin core domain [10]. And they are involved in many vital processes of cells including oxidative stress response, cell cycle regulation, aging, cell differentiation, energy metabolism, genomic stability, and tumorigenesis [11, 12]. Among them, Sirt1 deacetylates FOXO family members, p300, nuclear factor-*κ*B (NF-*κ*B), p53, and histones [12, 13], which regulate cell survival and cellular stress response. It also regulates Peroxisome Proliferator-Activated Receptor-*γ*, mTOR, and 5′-AMP-dependent kinase (AMPK), which play roles in cellular energy metabolism and autophagy [11, 12]. SIRT1 is already known related to many metabolic diseases, such as obesity [14–16], diabetes [17–19], and nonalcoholic fatty liver [19–21]. And in respect of cancer, SIRT1 has been reported to be involved in breast cancer [22–24], ovarian cancer [25–27], cervical cancer [28–30], gastric cancer [31–33], and prostate cancer [34–36]. But its role in bladder cancer remains largely unknown.

Recent studies of our group based on a transcriptome analysis using human bladder cancer tissues compared with normal bladder tissues [37–39], indicating a close correlation of Sirtuin family, PPAR signaling pathway, cell cycle regulation, ROS metabolism, and FOXO signaling pathway in BCa. Therefore, this study aims to investigate the effect of SIRT1 in BCa and the underlying mechanism.

## 2. Materials and Methods

### 2.1. Ethical Statement for Human Bladder Tissue Samples

Human bladder cancer tissue samples (*n* = 19) and human paracancerous tissues (*n* = 19) were all collected from patients suffering bladder cancer in surgery. And all normal bladder tissue samples (*n* = 4) were obtained from donors who died from accident. The samples were either stored in liquid nitrogen for later RNA isolation or fixed in 4% concentration paraformaldehyde (PFA) for the followed immunofluorescence staining analysis. The Ethics Committee at Zhongnan Hospital of Wuhan University approved the experiments using human bladder tissue samples for RNA and immunofluorescence staining analyses (approval number: 2015029, see Supplementary Material available online at https://doi.org/10.1155/2017/3781904). All methods used for human bladder tissue samples were performed in accordance with the approved guidelines and regulations. Written informed consent was obtained from all subjects.

### 2.2. Human Bladder Cancer Cells

Human bladder cancer cell line EJ (contaminated by T24 as per “http://iclac.org/databases/cross-contaminations/”) was from the Procell Co. Ltd. (carcinoma, Cat. #CL-0274) in Wuhan, China. And human bladder cancer cell line T24 (transitional cell carcinoma, Cat. #SCSP-536) was friendly provided by the Stem Cell Bank, Chinese Academy of Sciences in Shanghai, China. Both T24 and EJ (contaminated by T24 as per “http://iclac.org/databases/cross-contaminations/”) cells were cultured in a humidified atmosphere consisting of 95% air and 5% CO_2_ at 37°C in RPMI-1640 medium (Gibco, China) with 10% fetal bovine serum (FBS) (Gibco, Australia) and 1% penicillin G sodium/streptomycin sulphate inside.

### 2.3. RNA Expression Analyses

#### 2.3.1. Total RNA Isolation from Bladder Cells and Tissues

We isolated total RNA from cultured bladder cells and collected bladder tissues using Qiagen RNeasy Mini Kit (Cat. #74101, Qiagen Ltd., Germany), combined with QIAshredder from Qiagen (Cat. #79654, Qiagen Ltd., Germany), using a centrifuge (Cat. #5424, Eppendorf Ltd., Germany) according to the manufacturer's protocol. DNase I (RNase-Free DNase Set, Cat. #79254, Qiagen Ltd., Germany) was used to remove contamination of gDNA from the RNA samples. Concentration of isolated RNA was measured with NanoPhotometer (Cat. #N60, Implen Ltd., Germany).

#### 2.3.2. Microarray Analysis of mRNA Isolated from Human Bladder Tissues

As previously reported by Cao et al. [37], Wang et al. [38], and Qian et al. [39] from our group, a transcriptome analysis was established by using three human BCa versus three normal bladder tissues. Briefly, biotinylated cDNA were prepared from 250 ng total RNA using the Ambion® WT Expression Kit. Then, 5.5 *μ*g cDNA were hybridized on GeneChip Human Transcriptome Array 2.0 (16 h at 45°C) in Hybridization Oven 645. GeneChips were then washed and stained in the Affymetrix Fluidics Station 450 and scanned by Affymetrix® GeneChip Command Console (AGCC), installed in GeneChip® Scanner 3000 (7G). Data were analyzed by Robust Multichip Analysis (RMA). BCa related genes and pathways were analyzed by using Gene ontology (GO) and Go-map network analysis based on the Gene Cloud of Biotechnology Information software (GCBI System, Shanghai, China) (https://www.gcbi.com.cn) [40]. Thereafter, the gene list was subjected to the Database for Annotation, Visualization and Integrated Discovery (DAVID) [41] for annotation and overrepresentation analysis of the genes involved FOXO signaling pathway (map04068, KEGG pathway image, Kanehisa Laboratories, Japan [42, 43]). The microarray data was uploaded to the Gene Expression Omnibus (GEO) database with accession number: GSE76211. All data are MIAME compliant.

#### 2.3.3. Reverse Transcription and Quantitative Real-Time PCR (qRT-PCR)

Reverse transcription was conducted on an iCycler (Cat. #CFX Connect, Bio-Rad Ltd., USA) with ReverTra Ace qPCR RT Kit (Toyobo Ltd., China) using 1 *μ*g of total RNA mentioned above as template strand. Real-time polymerase chain reactions (qRT-PCR) were performed with iQTM SYBR® Green Supermix (Bio-Rad Ltd., China) in a reaction system of 20 *μ*l total volume using 1 *μ*g of cDNA. Before qRT-PCR all primers had been tested for optimal annealing temperatures with gradient PCRs. Primer sequences and annealing temperatures used in qRT-PCR are listed in Table 1. Values of GAPDH were used for normalizing amplification. To be more scientific, we used relative gene abundance for further statistical analyses: Δct = ct_target  gene_ − ct_GAPDH_, for BCa cells ΔΔct = Δct_siRNA-treated_ − Δct_siRNA-untreated_, for bladder tissues ΔΔct = Δct_BCa  patients_ − Δct_paracancerous  tissues_, and relative gene abundance = 2^−ΔΔct^ (ct = threshold cycle).

### 2.4. Cell Culture Experiments

#### 2.4.1. Knockdown of* SIRT1* in the BCa Cells

*Negative-control-siRNA *and three distinct* SIRT1*-target specific small interfering RNA* (siRNA)* were all synthesized by ViewSolid Ltd. in Beijing, China. The sequences of* siRNA* mentioned are listed in Table 2. These RNA were used to transfect BCa cell lines with LipoJet™ (SignaGen Ltd., China), according to the manufacturer's protocol. All transfected cells were cultured for another 48 h after transfection before being submitted in following tests or harvested for isolation of RNA or protein.

#### 2.4.2. ROS Detection by Staining with DCFH-DA

To measure the ROS level in the BCa cells, we used the fluorescent probe 2′,7′-dichlorofluorescin diacetate (DCFH-DA, Sigma-Aldrich Ltd., USA) for staining. 48 h after transfection, the BCa cells were harvested and centrifuged; then 1 ml serum-free medium containing 10 *μ*M DCFH-DA was added (Sigma-Aldrich Ltd., USA). After another incubation at 37°C for 30 min in dark, the BCa cells were washed by three times to wash away the extracellular DCFH-DA as fully as possible. Then the cells were submitted to flow cytometry for ROS analysis. For microscope photographing, serum-free medium containing both DCFH-DA and DAPI was added to the BCa cells slides which were then incubated for 30 min at the room temperature in the dark and washed three times. Microscope photographs were taken using a fluorescence microscope (Cat. #IX73, Olympus Ltd., Japan).

#### 2.4.3. Transwell Migration Assay

24-well plate transwell chamber system with 8.0 *μ*m pore size (Corning Ltd., USA) was used for transwell assy. BCa cells cultured for 48 h after transfection were harvested, centrifuged, and resuspended in serum-free medium and before being diluted to a certain density: 1.33 × 10^4^ cells per 100 *μ*l for EJ (contaminated by T24 as per “http://iclac.org/databases/cross-contaminations/”) and 2 × 10^4^ cells per 100 *μ*l for T24 cells. 300 *μ*l of suspension liquid was added to upper chamber insert, while the lower chamber was filled with 600 *μ*l of 10% FBS medium. After incubation for 24 h at 37°C, we used cotton swabs to scrub away the cells in the upper insert, and cells that migrated to the lower side were fixed by 4% PFA and stained by 0.1% crystal violet. After washing away crystal violet and air drying, the chambers were placed under an inverted phase contrast microscope (Cat. #DMI 1, Leica Ltd., Germany) to take pictures and count the migrated cells.

#### 2.4.4. MTT Assay

48 h after transfection, the BCa cells were seeded in 96-well plates (3,000 cells per 200 *μ*l medium) to be cultured for another 1–5 days. Every 24 h one plate was measured during the 5 days. When measuring, 20 *μ*l MTT (5 mg/ml) was added to each well before measured plate was incubated for another 4 h at 37°C, and then the medium was removed, formazan precipitate was dissolved in 150 *μ*l DMSO, and absorbance was measured at 490 nm with a microplate reader (Cat. #SpectraMax M2, Molecular Devices Ltd., USA).

#### 2.4.5. Clonogenic Forming Experiment

After transfection for 48 h, distinct BCa cells were seeded in 6-well plates (800 cells per well) and grew into colonies for approximately 14 days. Then colonies were fixed by 4% PFA for 30 min, stained with crystal violet for 30 min, washed with PBS, naturally dried, counted, and photographed.

#### 2.4.6. Flow Cytometry Analysis for Alterations of Cell Cycle and Apoptosis

For cell cycle analysis, the BCa cells were transfected by* siRNA* for 48 h and then harvested and centrifuged. Thereafter, the cells were washed by PBS for three times and resuspended with 1x DNA staining solution containing propidium iodide and permeabilization solution (Multisciences Ltd., China) in the dark. After another incubation (37°C for 30 min), cells were analyzed by flow cytometer (Cat. #FC500, Beckman Ltd., USA). Apoptosis analysis was assessed with the FITC Annexin V Apoptosis Detection Kit I (BD biosciences Ltd., USA), according to the manufacturer's instructions and analyzed by the flow cytometry analysis.

#### 2.4.7. TUNEL Assay

After 48 h transfection, coverslips with BCa cells were fixed by 4% PFA for 30 min at room temperature and washed three times by ice-cold PBS, continuously incubated with 0.1% Triton X-100 for 2 min, and washed by PBS for three times. Then, apoptotic cells were measured by the TdT-mediated dUTP-biotin nick end labeling test (TUNEL, Roche Applied Science Ltd., Germany), according to the manufacturer's instructions. Nuclei were stained by 1 *μ*M DAPI for 20 min at room temperature. Images were taken using the fluorescence microscope.

### 2.5. Protein Analyses

#### 2.5.1. Isolation of Total Protein from BCa Cells

The RIPA buffer with protease inhibitor and phosphatase inhibitor (Sigma-Aldrich Ltd., USA) was used to lyse and sonicate BCa cells on ice for 30 min and then centrifuged at 12,000*g* for 15 min to collect supernatant. The density of protein was measured by the Bradford protein assay (Bio-Rad Ltd., Germany).

#### 2.5.2. Western Blot and Immunoprecipitation Analysis

For the following Western blot analysis, total protein was separated by electrophoresis in 10–12.5% SDS-PAGE and then transferred to the PVDF membrane (Millipore Ltd., USA). Membranes were then blocked in 5% fat-free milk and continuously incubated with primary antibodies (Table 3) at 4°C for overnight and secondary antibodies (Table 4) for 2 h at room temperature. Bands were visualized using an enhanced chemiluminescence (ECL) kit (Bio-Rad Ltd., USA) and detected by ChemiDoc XRS^+^ Imaging System (Bio-Rad Ltd., USA).

The immunoprecipitation analysis for acetylation level of FOXO3a was done according to Frazzi et al. [44]. Briefly, total cell lysates were prepared with lysis buffer and cleared by centrifugation. Then, 200 *μ*l of cell lysates was incubated with anti-FOXO3a antibody and rotated at 4°C for overnight. The samples were continually incubated with protein A agarose for 2 hours. After washing the immunoprecipitated produce was eluted, loaded on 10% polyacrylamide gels, and performed by SDS-PAGE, as well as detected by the ECL kit. The intensity of bands was measured with the ancillary software of ChemiDoc XRS^+^ Imaging System (Bio-Rad Ltd., USA), named Image Lab (version 5.1, build 8). To compare the acetylation level of FOXO3a, we calculated the relative acetylation rate of FOXO3a: the relative acetylation rate = intensity of anti-acetyl Lysine/intensity of total FOXO3a band. The intensity of total FOXO3a was used as a loading control and its rate of NC group was normalized to 1.

#### 2.5.3. Immunofluorescence Staining for Human Bladder Tissue Samples

The bladder tissue samples were fixed by 4% PFA containing 2% sucrose in PBS at 4°C for overnight and embedded into paraffin (Paraplast, Sigma-Aldrich Ltd., USA) using a tissue processor (Cat. #STP 120, Thermo Fisher Scientific Ltd., UK). Paraffin sections (4 *μ*m) were cut with a rotation microtome (Cat. #HM325, Thermo Fisher Scientific Ltd., Germany). The sections were serially incubated with primary antibody and Cy3-labeled or FITC- labeled secondary antibody in humidified atmosphere (Tables 3 and 4). Nuclei were labeled with DAPI (2 *μ*g/ml). Immunofluorescence staining images for paraffin sections were analyzed by the fluorescence microscope.

#### 2.5.4. Immunofluorescence Analysis for BCa Cells

Coverslips with BCa cells were washed three times with ice-cold PBS and fixed by 4% PFA for 30 min. Cells were then treated with 0.1% Triton X-100 solution and blocked using normal goat serum for 30 min at room temperature. Afterwards, the cells were incubated with the indicated primary antibody (Table 3) at the proper dilution for 2 h at room temperature, washed with PBS for three times, and incubated with Cy3-labeled or FITC labeled secondary antibody (Table 4) for 1 h. Nuclei were stained by 1 mM TOTO-3 iodide for 10 min at room temperature. Immunofluorescence staining was analyzed by the fluorescence microscope.

### 2.6. Statistical Analyses

All data described as mean ± SD form is from three or more independent experiments. Two-tailed Student's paired and unpaired *t*-tests were used to evaluate the statistical significance of the data. All of the statistical analyses were performed with SPSS 16.0. Statistical significance was set at probability values of *p* < 0.05.

## 3. Results 

### 3.1. Upregulation of SIRT1 in BCa Tissues Compared with Paracancerous Tissues and Normal Bladder Tissues

**Figure 1:**
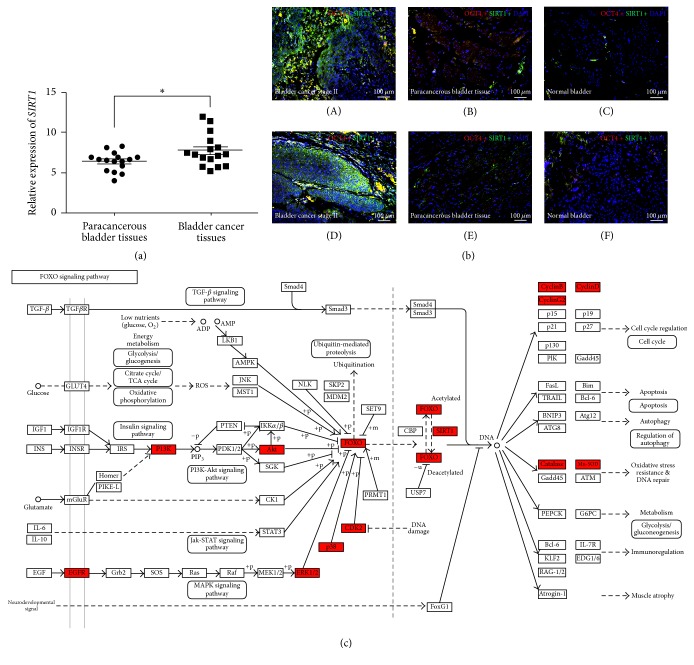
*SIRT1 is strongly upregulated in the BCa tissues compared with both paracancerous tissues and normal bladder tissues*. (a) qRT-PCR analysis exhibited that the gene expression of* SIRT1* in bladder cancer tissues was significantly higher than the matched paracancerous tissues. The value of GAPDH was used as an internal control. ^*∗*^*p* < 0.05. (b) Representative result of double immunofluorescence staining of SIRT1 (green) in bladder cancer tissues (A, D), paracancerous tissues (B, E), and normal bladder tissues (C, F). The OCT4 (red) was stained as a marker of BCa cells. Nuclei (blue) were stained by DAPI. The scale bar for (b) is 100 *μ*m. (c) Overrepresentation analysis using microarray raw data and DAVID database suggested that the FOXO signaling pathway and related genes were affected (red) in the BCa tissues (FOXO signaling pathway, map04068, KEGG pathway image, copyright permission obtained from KEGG).

qRT-PCR analysis was performed to evaluate the expression of* SIRT1 *gene, indicating significant upregulation in the BCa tissues compared with the paired paracancerous tissues (*n* = 16, *p* < 0.05, Figure 1(a)). Double immunofluorescence staining showed that, in the BCa tissues, SIRT1 protein was strongly increased in the OCT4-positive cells (Figure 1(b)), which has been suggested to be a potential biomarker for BCa [37], whereas the corresponding paracancerous bladder tissues and the normal bladder tissues exhibited slight staining of both OCT4 and SIRT1 protein (Figure 1(b)), consisting with the qRT-PCR result. Overrepresentation analysis using microarray raw data [37–39] and DAVID database revealed that, in the BCa tissues, increased* SIRT1* expression could affect oxidative stress resistance, cell cycle regulation, and energy metabolism, via FOXO signaling pathway (Figure 1(c)).

### 3.2. *SIRT1* Deficiency Affected ROS Level in BCa Cells

**Figure 2:**
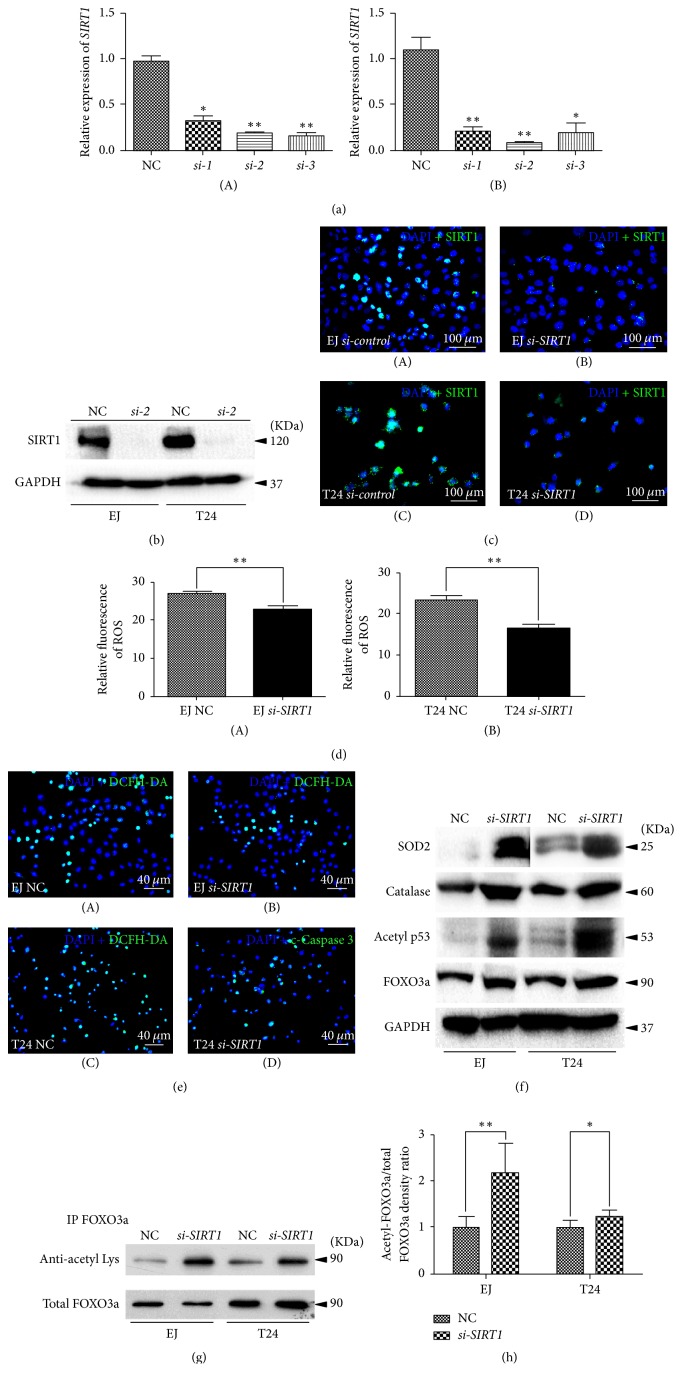
*Knockdown of SIRT1 in BCa cells induced alteration of ROS and related proteins*. (a) qRT-PCR was used to testify the KD efficiency by using different* siRNA *to knockdown* SIRT1 *in T24 (A) and EJ (B) (contaminated by T24 as per “http://iclac.org/databases/cross-contaminations/”) bladder cancer cells. All shown values were mean ± SD of three measurements and repeated three or more times, ^*∗*^*p* < 0.05 and ^*∗∗*^*p* < 0.01. (b) Western blot bands of SIRT1 and GAPDH in EJ (contaminated by T24 as per “http://iclac.org/databases/cross-contaminations/”) and T24,* SIRT1* deficiency BCa cells compared with NC BCa cells. The GAPDH abundance was used as an internal control. (c) Representative immunofluorescence staining of SIRT1 (green) in the BCa cells after* SIRT1-target-specific-siRNA *treatment (KD) (B, D), compared with* control-siRNA *treatment (NC) (A, C). Nuclei (blue) were stained by DAPI. The scale bar for (c) is 100 *μ*m. (d) Statistical analysis of relative fluorescence of ROS in the NC and* si-SIRT1* transfected EJ (A) (contaminated by T24 as per “http://iclac.org/databases/cross-contaminations/”) and T24 (B) cells. ^*∗∗*^*p* < 0.01. (e) Representative DCFH-DA staining of ROS (green) in the BCa cells with* si-SIRT1 *treatment (B, D) versus* control-siRNA *treatment (NC) (A, C). Nuclei (blue) were stained by DAPI. The scale bar for (e) is 40 *μ*m. (f) Western blot analyses of antioxidant enzymes (SOD2, Catalase) acetylated p53 and total FOXO3a in the NC and* si-SIRT1* transfected EJ (contaminated by T24 as per “http://iclac.org/databases/cross-contaminations/”) and T24 cells (cell types,* siRNA* treatment, and protein masses were indicated). The GAPDH was used as a loading control. 10–30 *μ*g of total protein were loaded per lane. (g) Immunoprecipitation analysis of antiacetylated Lysine and total FOXO3a in the NC and* si-SIRT1* transfected EJ (contaminated by T24 as per “http://iclac.org/databases/cross-contaminations/”) and T24 cells. (h) Statistical analysis of relative acetylation rate of FOXO3a by measurement of the band intensity of three independent Western blot experiments, calculated as relative acetylation rate of FOXO3a = intensity of antiacetylated Lysine/intensity of total FOXO3a. The intensity of total FOXO3a was used as control and its rate of NC group was normalized to 1. ^*∗*^*p* < 0.05; ^*∗∗*^*p* < 0.01.

A cell model of* SIRT1 *deficiency was established by* siRNA*-transfection in the BCa cells. The BCa cells were transfected by three distinct* SIRT1-target-specific-siRNA (si-1, si-2, and si-3)* and* negative-control-si*RNA (NC) (sequences are listed in Table 1). Efficiency of knockdown was validated by qRT-PCR 48 h after transfection (Figure 2(a)). Considering qRT-PCR result in both cell lines overall, we used* si-2* to perform the following experiments. Knockdown rate was 80.4% in EJ (contaminated by T24 as per “http://iclac.org/databases/cross-contaminations/”) and 86.8% in T24 cells. We noticed a significant downregulation of SIRT1 protein abundance was observed by Western blot analysis (Figure 2(b)) and immunofluorescence staining (Figure 2(c)), indicating transfection by the* SIRT1-target-specific-siRNA si-2* could reduce SIRT1 at both transcriptional and translational levels in both BCa cell lines.

SIRT1 is reported to play a role in the metabolism of the reactive oxygen species (ROS) in cells [36–38], but how SIRT1 affected ROS metabolism in bladder cancer cells has not been reported yet. Therefore, we used DCFH-DA to stain the* si-SIRT1 *group and NC group before flow cytometry analysis. The results suggested that BCa cells lacking* SIRT1* exhibited a reduced level of ROS production than the NC group (Figure 2(d)). Consistently, ROS staining using fluorescence staining showed a similar result (Figure 2(e)), accompanied by increasing of distinct antioxidant enzymes (Catalase and SOD2, Figure 2(f)). Moreover, our results suggested that the central transcription receptors (FOXO3a, PPAR*γ*, and acetylated p53) were strongly upregulated in the* si-SIRT1 *group (Figure 2(f)).

### 3.3. Acetylation Level of FOXO3a Was Induced in the BCa Cells with Downregulated* SIRT1*

Since no effective antibody for acetylated FOXO3a is available, we used immunoprecipitation experiment to test the acetylation level of FOXO3a (Figure 2(g)). By using the intensity of total FOXO3a as a loading control, the relative acetylation rate of FOXO3a was statistically analyzed (Figure 2(h)). The results suggested that the acetylation level of FOXO3a in the downregulated* SIRT1* group was significantly increased.

### 3.4. Knockdown of* SIRT1* Inhibited Proliferation and Viability in BCa Cells and Triggered Cell Cycle Arrest at G0/G1 Phase

**Figure 3:**
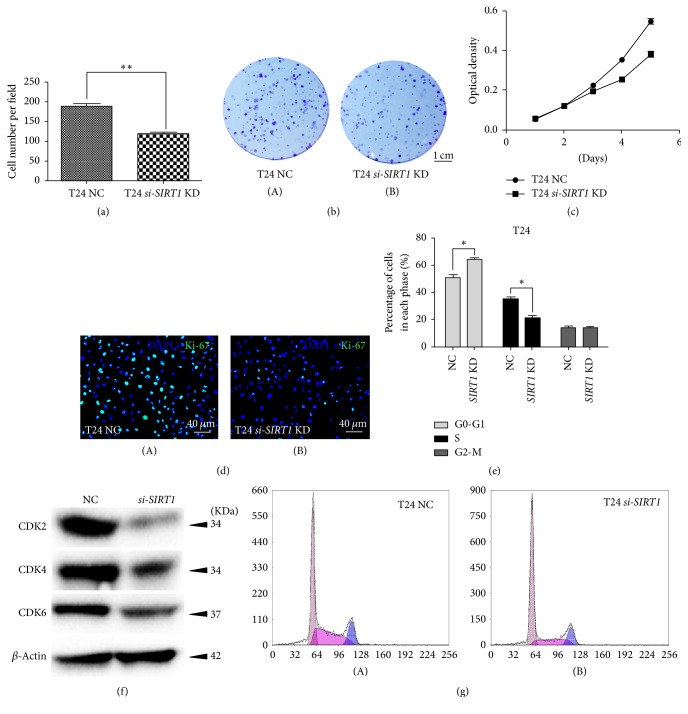
*Downregulation of SIRT1 repressed BCa cell proliferation and induced cell cycle arrest*. (a) Clone number in each well was counted and statistically analyzed in the clonogenic survival assay. ^*∗∗*^*p* < 0.01. (b) Clonogenic survival assay revealed cell survival of BCa cells after treatment of* SIRT1-target-specific-siRNA* (*SIRT1 *KD) and* control-siRNA* (NC), cultured in 6-well plates for 14 days. (c) MTT assay was used to measure the viability of BCa cells treated by* SIRT1-target-specific-siRNA *(*SIRT1 *KD, line linking squares) and* negative-control-siRNA* (NC, line linking circles). All shown values were mean ± SD of three measurements and repeated three times with similar results, ^*∗*^*p* < 0.05. (d) Cell proliferation of BCa cells treated by* SIRT1-target-specific-siRNA *(B) and* negative-control-siRNA *(A) was assayed by Ki-67 staining (green). Nuclei (blue) were stained by DAPI. (e) Statistical analysis of percentages (%) of BCa cell populations at different stages of cell cycles. All shown values were mean ± SD of three measurements and repeated three times with similar results. ^*∗*^*p* < 0.05. (f) Western blot analysis of proteins involved in G0-G1 cell cycle regulation (CDK2, CDK4, and CDK6) in the BCa cells. *β*-Actin abundance was used as a control. (g) Flow cytometry analysis result for BCa cells treated with* negative-control-siRNA *(A) and* SIRT1-target-specific-siRNA *(B) for 48 h. The scale bar for (b) is 1 cm and for (d) is 40 *μ*m.

Clonogenic survival assay revealed that the ability of clone formation was decreased in the* si-SIRT1* cells by 36.5%, comparing with the NC group (Figures 3(a) and 3(b)). Moreover, MTT assay revealed that the* si-SIRT1*-treated BCa cells grew significantly slower than the* negative-control-si*RNA-treated group (Figure 3(c)), consisting with strongly reduced Ki-67 positive cells in the* si-SIRT1* group (Figure 3(d)), which could be used as an indicator for proliferation [45].

Flow cytometry analysis of cell cycle indicated that there was higher percentage of cells at G0/G1 phase in* si-SIRT1* group than in NC group while it was on the contrary at S phase (Figures 3(e) and 3(g)), which suggested that* SIRT1* deficiency induced cell cycle arrest at G0/G1 phase. In addition, Western blot result suggested proteins involved in regulating G0/G1 phase (CDK2/4/6) were considerably decreased in* si-SIRT1 *group (Figure 3(f)).

### 3.5. Downregulation of* SIRT1* Inhibited Migration in BCa Cells

**Figure 4:**
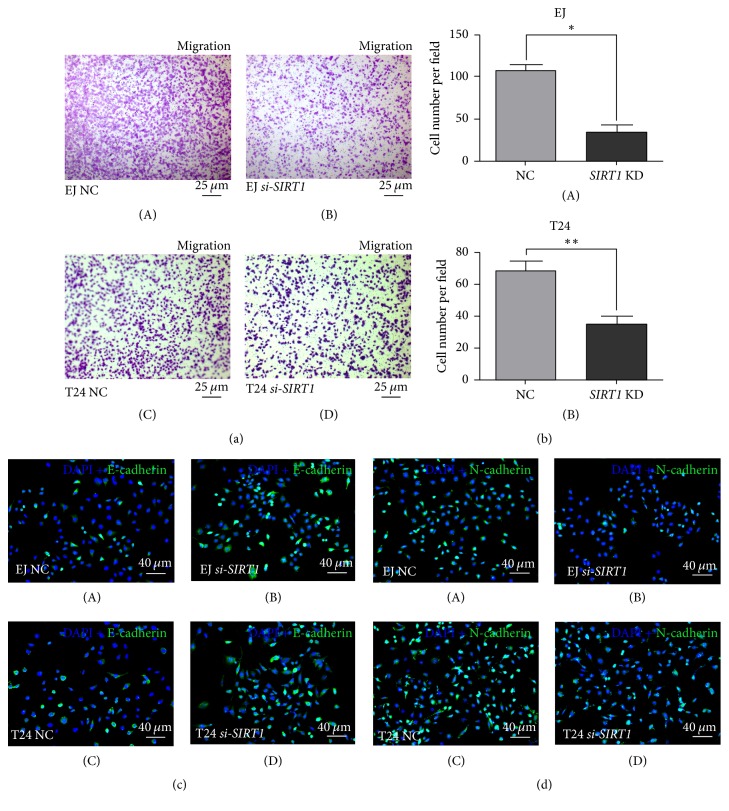
*SIRT1 deficiency inhibited migration of BCa cells*. (a) Transwell migration assay was conducted to measure the migration ability of NC group cells (A, C) and* si-SIRT1 *group cells (B, D) in EJ (contaminated by T24 as per “http://iclac.org/databases/cross-contaminations/”) and T24 for 48 h after transfection. (b) BCa cells in transwell migration assay were counted on microscope and statistically analyzed. All shown values were mean ± SD of three measurements and repeated three times with similar results. ^*∗*^*p* < 0.05; ^*∗∗*^*p* < 0.01. (c) Representative result of immunofluorescence staining of E-cadherin (green) in the BCa cells after* SIRT1-target-specific-siRNA *treatment (KD) (B, D), compared with* control-siRNA *treatment (NC) (A, C). Nuclei (blue) were stained by DAPI. The scale bar for (D) is 40 *μ*m. (d) Representative result of immunofluorescence staining of N-cadherin (green) in the BCa cells after* SIRT1-target-specific-siRNA *treatment (KD) (B, D), compared with* control-siRNA *treatment (NC) (A, C). Nuclei (blue) were stained by DAPI. The scale bars for (a) are 25 *μ*m and for (c-d) are 40 *μ*m.

Cell migration was measured using transwell migration assay, and migration rate was significantly lower in* si-SIRT1* group than the NC group both in EJ (contaminated by T24 as per “http://iclac.org/databases/cross-contaminations/”) and in T24 (Figures 4(a) and 4(b)). Affected by the transfection of* SIRT1-target-specific-siRNA*, the migration rate declined by 67.5% (*p* < 0.05) in EJ (contaminated by T24 as per “http://iclac.org/databases/cross-contaminations/”) and 48.5% in T24 (*p* < 0.01). Furthermore, the protein abundances of E-cadherin and N-cadherin, involved in the epithelial-mesenchymal transition (EMT) process, were affected by reduction of* SIRT1*. Our results showed that the E-cadherin level was obviously higher and the N-cadherin was lower in* si-SIRT1 *group (Figures 4(c) and 4(d)), suggesting that activity of EMT process was reduced by* SIRT1* deficiency.

### 3.6. Lower Level of* SIRT1* Expression Had No Significant Effect on Apoptosis in BCa Cells

**Figure 5:**
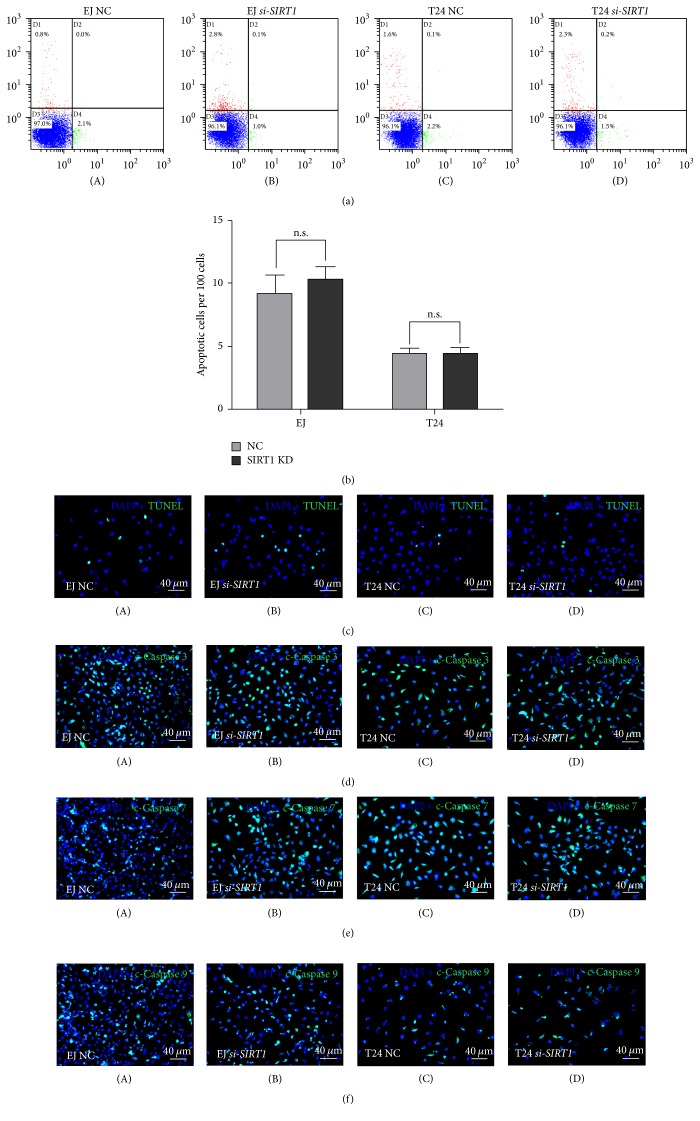
*Decreased SIRT1 could not lead to BCa cell apoptosis*. (a) The human bladder cancer cells EJ (contaminated by T24 as per “http://iclac.org/databases/cross-contaminations/”) and T24 were transfected with* SIRT1-target-specific-siRNA *(B, D) and* control-siRNA *(A, C) for 48 h. BCa cells were stained with Annexin V/PI and apoptosis was measured by flow cytometry. (b) Statistical analysis of TUNEL-test revealed no significant (n.s.) increase of apoptotic cell rate in either EJ (contaminated by T24 as per “http://iclac.org/databases/cross-contaminations/”) or T24 cells after* SIRT1-target-specific-siRNA *(*SIRT1 *KD) treatment. (c) TUNEL-test to detect apoptotic cells (green) in* control-siRNA*-treated BCa cells (A, C) and* SIRT1-target-specific-siRNA-*treated BCa cells (B, D). Nuclei (blue) were stained by DAPI. (d, e, f) Representative immunofluorescence images of cleaved-Caspases 3, 7, and 9 (green) in the T24 and EJ (contaminated by T24 as per “http://iclac.org/databases/cross-contaminations/”) cells after* SIRT1-target-specific-siRNA *treatment (KD) (B, D), compared with* control-siRNA *treatment (NC) (A, C). Nuclei (blue) were stained by DAPI. The scale bars for (c–f) are 40 *μ*m.

We also performed flow cytometry analysis to investigate apoptosis alternation of* SIRT1* deficiency. But after multiple repeated tests, the result yet suggested no significant alteration between* si-SIRT1 *group and NC group (Figure 5(a)). To make further affirmation, we conducted TUNEL assay, and the result suggested no significant alteration either (Figure 5(b)). Consistently, in immunofluorescence staining, no significant (n.s.) alteration of the apoptosis related proteins cleaved-Caspases 3, 7, and 9 in* si-SIRT1 *group was observed (Figures 5(d)–5(f)).

## 4. Discussion

In our previous studies [37–39] based on comprehensive transcriptome analysis using human bladder cancer tissues compared with normal bladder tissues, we found that* SIRT1 *played a vital role in tumorigenesis and further development of human bladder cancer (BCa).* SIRT1 *is a crucial gene in process of aging [9], energy metabolism, and autophagy [11], but its role in BCA remains largely unknown. Our further investigation in human bladder tissue samples at a larger scale revealed that SIRT1 possessed an overexpression in human bladder cancer tissues than in paracancerous tissues or normal bladder tissues, at both transcriptional and protein levels (Figure 1). Therefore, overrepresentation analysis and DAVID database (Figure 1) revealed that* SIRT1* could interfere FOXO signaling pathway to affect oxidative stress resistance, cell cycle regulation, and energy metabolism in the BCa tissues.

FOXO proteins constitute a family of transcription factors that play important role in regulating the expression of genes involved in cell growth, proliferation, differentiation, and longevity. FOXO3a is a key submember in the FOXO family involved in AKT/FOXO3a/*β*-catenin pathway [39], regulating cell cycle, oxidative stress response, and apoptosis [46–48]. In the downregulated* SIRT1* BCa cells, total and acetylated FOXO3a were strongly induced (Figure 2), suggesting the correlated cell cycle regulation and antioxidant response were affected. These results were similar as reported by Frazzi et al. [44], which indicated that by using an inhibitor of SIRT1 (resveratrol) the acetylation level of FOXO3a was strongly upregulated in the Hodgkin lymphoma cells.

Indeed, in our* SIRT1* deficiency cell model, we observed a significantly reduced ROS production (Figure 2). As we know, the role that ROS plays in cancer biology is quite complicated, which can be seen as a double-edged sword. A modest level of ROS is essential for tumorigenesis, whereas an excessive level would suppress tumors [49, 50]. ROS can serve as a signal of either apoptosis or survival, which is determined by dosage, type, duration, and site of ROS production [51]. As Lin et al. reported, ROS produced in mitochondria could be the major source of ROS production and therefore lead to BCa cells apoptosis [52]. Therefore, the alteration of mitochondrial SOD2 was analyzed, which clears mitochondrial ROS, thus protecting cells from cell death [53]. Consistently, our result revealed an obvious upregulation of SOD2 protein (Figure 2), which may be responsible for no significant increase of BCa cells apoptosis observed (Figure 5). Another key enzyme involved in antioxidant response is Catalase, which has been reported to protect chromosomes against ionizing radiation [53] or oxidative damage [54]; therefore it suppresses cell death. And it also showed an increase in our study (Figure 2(f)), which may be another reason to explain that no significant increase of apoptosis occurred in the* si-SIRT1* treated BCa cells (Figure 5).

Moreover, we observed a significantly induced cell cycle arrest at G0/G1 phase (Figure 3), followed by downregulation of proteins involved in G0/G1 to S phase progression (CDK2/4/6). Cyclin-dependent kinases (CDKs) are a family of protein kinases playing vital roles in regulating cell cycle [55]. Among them CDK4 and CDK6 are responsible for helping cells getting out from G0 phase and into G1 phase [56, 57]; meanwhile CDK2 plays its role in G1 phase [58]. Downregulation of CDK2/4/6 could lead to cell cycle arrest at G0/G1 phase [59], which may explain our result of flow cytometry analysis (Figure 2).

We highly suspect that the highly activated antioxidant response may play a vital role in the process avoiding the BCa cells with* SIRT1 *deficiency from apoptosis. However, further studies are needed for the detailed mechanism.

For further discussion, it is reported that the overexpression of Thromboxane-A2 isoform-*β* receptor* (TPβ)* plays an important role in the process of human bladder cancer, and TP agonist decreased acetylation of FOXO3 via upregulation of* SIRT1 *in the bladder cancer cell line UMUC3 [60]. However, no significant alteration of* TPβ* between human normal bladder and bladder cancer tissue was found in our microarray. It may suggest that there are other pathways regulating* SIRT1* expression in bladder cancer. Moreover, as reported by Lin et al. [52], capsaicin inhibits tumor-associated NADH oxidase (tNOX) and SIRT1, thus changing multiple phenotypes of bladder cancer cells including apoptosis, cell cycle progression, and cell migration. In contrast, in our study we directly knocked down* SIRT1*, which could help us focus on the effect of* SIRT1 *downregulation, avoiding the interference of other factors.

## 5. Conclusions

Our study for the first time suggested that* SIRT1* deficiency in bladder cancer cells could suppress proliferation and ROS production, as well as induce cell cycle arrest, possibly via the FOXO3a-mediated pathways.

## Supplementary Material

The uses and analysis of human bladder tissues were approved by the Ethics Committee at Zhongnan Hospital of Wuhan University with approval number 2015029.

## Figures and Tables

**Table 1:** List of primers for qRT-PCR.

Gene	Symbol	Forward primer (5′-3′)	Reverse primer (5′-3′)	Annealing temperature (°C)	Length (bp)
Sirtuin 1	*SIRT1*	5′-TAGCCTTGTCAGATAAGGAAGGA-3′	5′-ACAGCTTCACAGTCAACTTTGT-3′	58	160
Glyceraldehyde-3-phosphate dehydrogenase	*GAPDH*	5′-ACAACTTTGGTATCGTGGAAGG-3′	5′-GCCATCACGCCACAGTTTC-3′	56	101

**Table 2:** List of *siRNA.*

Mark name	Suppressed gene	Sense sequence (5′-3′)	Antisense sequence (5′-3′)	Supplier
NC	Negative control	UUCUCCGAACGUGUCACGUTT	ACGUGACACGUUCGGAGAATT	Viewsolid Biotech, Beijing
*si-1*	SIRT1	GGAAAUAUAUCCUGGACAATT	UUGUCCAGGAUAUAUUUCCTT	Viewsolid Biotech, Beijing
*si-2*	SIRT1	GCAACUAUACCCAGAACAUTT	AUGUUCUGGGUAUAGUUGCTT	Viewsolid Biotech, Beijing
*si-3*	SIRT1	GCUGAUGAACCGCUUGCUATT	UAGCAAGCGGUUCAUCAGCTT	Viewsolid Biotech, Beijing

**Table 3:** List of primary antibodies.

Antigens	Species antibodies raised in	Dilution (IF)	Dilution (WB)	Supplier
Acetyllysine, acetylated KLH conjugates	Rabbit, polyclonal	—	1 : 500	Abcam, UK, Cat. #ab80178
Catalase, human	Rabbit, monoclonal	—	1 : 2,000	Abcam, UK, Cat. #ab76024
CDK2, human	Rabbit, monoclonal	—	1 : 2,000	Abcam, UK, Cat. #ab32147
CDK4, human	Rabbit, monoclonal	—	1 : 2,000	Abcam, UK, Cat. #ab124821
CDK6, human	Rabbit, monoclonal	—	1 : 1,000	Abcam, Cat. #ab124821
Cleaved caspase-3, human	Rabbit, monoclonal	1 : 200	—	Cell Signaling Technology, USA, Cat. #9664
Cleaved caspase-7, human	Rabbit, monoclonal	1 : 200	—	Cell Signaling Technology, USA, Cat. #8438
Cleaved caspase-9, human	Rabbit, monoclonal	1 : 200	—	Cell Signaling Technology, USA, Cat. #7237
FOXO3a, human	Rabbit, monoclonal	—	1 : 1,000	Abcam, UK, Cat. #ab53287
Ki-67, human	Rabbit, monoclonal	1 : 200	—	Novus Biologicals, USA, Cat. #NBP2-19012
OCT-4, human	Mouse, monoclonal	1 : 200	—	Novus Biologicals, USA, Cat. #NB110-90606
p53 (acetyl K370), human	Rabbit, monoclonal	—	1 : 1,000	Abcam, UK, Cat. #ab183544
PPAR*γ*, human	Rabbit, monoclonal	—	1 : 1,000	Abcam, UK, Cat. #ab45036
SIRT1, human	Rabbit, monoclonal	1 : 400	1 : 1,000	Cell Signaling Technology, USA, Cat. #9475
SOD2, human	Rabbit, monoclonal	—	1 : 1,000	Abcam, UK, Cat. #ab68155
*β*-Actin, human	Mouse, monoclonal	—	1 : 2,000	Santa Cruz Biotechnology Inc., Dallas, TX., USA, Cat. #sc-47778
E-Cadherin, human	Rabbit, monoclonal	1 : 200	—	Cell Signaling Technology, USA, Cat. #3195
N-Cadherin, human	Rabbit, monoclonal	1 : 200	—	Cell Signaling Technology, USA, Cat. #13116

**Table 4:** List of secondary antibodies and counterstaining of nuclei.

Secondary detection system used	Host	Method	Dilution	Supplier
Anti-mouse-IgG (H + L)-HRP	Goat	WB	1 : 10,000	Sungene Biotech, China, Cat. #LK2003
Anti-rabbit-IgG (H + L)-HRP	Goat	WB	1 : 10,000	Sungene Biotech, China, Cat. #LK2001
Anti-rabbit IgG (H + L), F(ab′)2 fragment (Alexa Fluor® 488 Conjugate)	Goat	IF	1 : 50	Cell Signaling Technology, USA, Cat. #4412
Anti-mouse IgG (H + L), F(ab′)2 fragment (Alexa Fluor® 555 Conjugate)	Goat	IF	1 : 50	Cell Signaling Technology, USA, Cat. #4408
Anti-goat IgG-FITC	Rabbit	IF	1 : 100	Boster Biological Technology, China, Cat. #BA1110
Anti-goat IgG-Cy3	Rabbit	IF	1 : 100	Boster Biological Technology, China, Cat. #BA1034
Hoechst 33342 nucleic acid staining (DAPI)	—	IF	1 : 750	Molecular Probes/Invitrogen, USA, Cat. #A11007
