# Supplementary material for: Knockdown of SIRT1 Suppresses Bladder Cancer Cell Proliferation and Migration and Induces Cell Cycle Arrest and Antioxidant Response through FOXO3a-Mediated Pathways
Source: Biomed Res Int. 2017 Sep 25;2017:3781904. doi: 10.1155/2017/3781904 (PMC5632854; doi:10.1155/2017/3781904)
Supplement: Supplementary file 1 — The uses and analysis of human bladder tissues were approved by the Ethics Committee at Zhongnan Hospital of Wuhan University with approval number 2015029. [file 3781904.f1.docx]

**Supplementary Material**


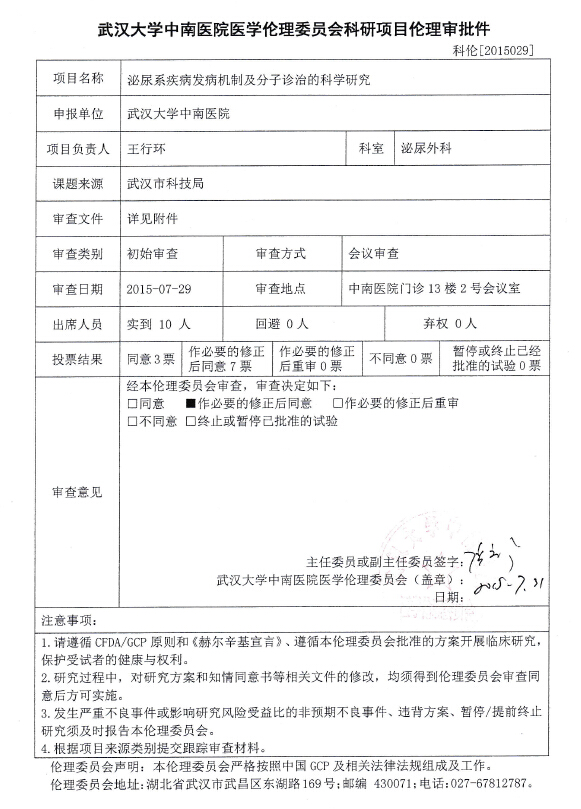


**Supplementary Material 1. Ethics Committee Approval.** The Ethics Committee at Zhongnan Hospital of Wuhan University approved the experiments of RNA and immunofluorescence staining analyses using human bladder cancer tissues and normal bladder tissues from donors by accidental death. Ethics Committee approval number: 2015029.
